# Retraction: The Patterns of Non-adherence to Medication in the Management of Cardiovascular Disease: A Descriptive Study

**DOI:** 10.7759/cureus.r184

**Published:** 2025-05-29

**Authors:** Anurag Agarwal, Shilpa Mannagudda Sandip, Amey Joshi, Arshitha Ashok

**Affiliations:** 1 General Surgery, Betsi Cadwaladr University Health Board, Bangor, GBR; 2 Community Medicine, Vydehi Institute of Medical Sciences and Research Centre, Bangalore, IND; 3 Internal Medicine, Sparrow Hospital-Michigan State University, East Lansing, USA; 4 Ophthalmology, Jagadguru Jayadeva Murugarajendra (JJM) Medical College, Davanagere, IND

This article has been retracted by the Editors-in-Chief at the request of the authors due to the use of a copyrighted scale without the necessary permissions. The figures depicting the copyrighted scale and questionnaire have been removed from the retracted article. All authors have agreed to the retraction.

